# Plasma Angiotensin II Is Increased in Critical Coronavirus Disease 2019

**DOI:** 10.3389/fcvm.2022.847809

**Published:** 2022-06-24

**Authors:** Rafael L. Camargo, Bruna Bombassaro, Milena Monfort-Pires, Eli Mansour, Andre C. Palma, Luciana C. Ribeiro, Raisa G. Ulaf, Ana Flavia Bernardes, Thyago A. Nunes, Marcus V. Agrela, Rachel P. Dertkigil, Sergio S. Dertkigil, Eliana P. Araujo, Wilson Nadruz, Maria Luiza Moretti, Licio A. Velloso, Andrei C. Sposito

**Affiliations:** ^1^Obesity and Comorbidities Research Center, University of Campinas, Campinas, Brazil; ^2^Department of Internal Medicine, School of Medical Sciences, University of Campinas, Campinas, Brazil; ^3^Department of Radiology, School of Medical Sciences, University of Campinas, Campinas, Brazil; ^4^School of Nursing, University of Campinas, Campinas, Brazil

**Keywords:** angiotensin converting enzyme 2, coronavirus, inflammation, lung hypertension, renin, lung, SARS-CoV-2, biomarker

## Abstract

Severe acute respiratory syndrome coronavirus 2 (SARS-CoV-2) employs angiotensin-converting enzyme 2 (ACE2) as its receptor for cell entrance, and studies have suggested that upon viral binding, ACE2 catalytic activity could be inhibited; therefore, impacting the regulation of the renin-angiotensin-aldosterone system (RAAS). To date, only few studies have evaluated the impact of SARS-CoV-2 infection on the blood levels of the components of the RAAS. The objective of this study was to determine the blood levels of ACE, ACE2, angiotensin-II, angiotensin (1–7), and angiotensin (1–9) at hospital admission and discharge in a group of patients presenting with severe or critical evolution of coronavirus disease 2019 (COVID-19). We showed that ACE, ACE2, angiotensin (1–7), and angiotensin (1–9) were similar in patients with critical and severe COVID-19. However, at admission, angiotensin-II levels were significantly higher in patients presenting as critical, compared to patients presenting with severe COVID-19. We conclude that blood levels of angiotensin-II are increased in hospitalized patients with COVID-19 presenting the critical outcome of the disease. We propose that early measurement of Ang-II could be a useful biomarker for identifying patients at higher risk for extremely severe progression of the disease.

## Introduction

Angiotensin-converting enzyme 2 (ACE-2) acts as the receptor for severe acute respiratory syndrome coronavirus 2 (SARS-CoV-2; 1) and viral binding and internalization are believed to impair ACE-2 catalytic activity, impacting the inactivation of angiotensin II (Ang-II; 2–4). It has been proposed that abnormal regulation of the renin-angiotensin-aldosterone system (RAAS) could explain cardiovascular and inflammatory instability, which play important roles in the severe progression of coronavirus disease 2019 (COVID-19; 5–7). This is further supported by the fact that hypertension, diabetes, and obesity, conditions that are related to abnormal regulation of the RAAS, are the main risk factors for the severe progression of COVID-19 (8, 9). In addition, a recent meta-analysis has shown that pharmacological inhibition of the RAAS is associated with reduced mortality of patients with COVID-19 (10). Nevertheless, there are still several questions that have not been properly answered in this field.

Angiotensin II is produced from angiotensin I (Ang-I) as a result of the catalytic action of angiotensin-converting enzyme 1 (ACE-1). Ang-II plays an important role in the regulation of blood volume, blood pressure, cardiac function, and electrolyte balance (11, 12). Moreover, increased Ang-II can lead to mitochondrial oxidative damage, reactive oxygen species overproduction, and increased interleukin-6 (IL-6); thus, boosting inflammation (13, 14). Ang-II is also involved in the regulation of insulin action, as both signaling systems interact at the intracellular level (15, 16). Therefore, determining the potential involvement of Ang-II in severe COVID-19 could provide advances in the diagnosis and treatment of the disease.

Currently, only few studies have measured the components of the RAAS in the blood of patients with COVID-19 and the results are mostly controversial (17–19). In this study, we determined the blood levels of the RAAS components in patients with severe progression of COVID-19. We showed that, at hospital admission, Ang-II was increased in the blood of patients that presented with an extremely severe progression of COVID-19. We propose that early measurement of Ang-II could be a useful marker for identifying patients at higher risk for extremely severe progression of the disease.

## Methods

### Study Cohort and Data Collection

This study was conducted at the Clinics Hospital at the University of Campinas in Brazil. Sample and data collection were performed within the study “*Safety and Outcomes Associated with the Pharmacological Inhibition of the Kinin–Kallikrein System in Severe COVID-19*” (20), a phase II, single-center, three-arm parallel-group, open-label, randomized clinical trial. Thirty patients diagnosed with COVID-19, as confirmed by real time-PCR analysis of nasopharyngeal swab samples, were enrolled from 22 April–14 June 2020. The study was approved by the ethics committee of the University of Campinas (protocol CAEE: 30227920.9.0000.5404), conducted according to the Declaration of Helsinki and Good Clinical Practice principles (ICH 1996) and registered with the Brazilian Clinical Trials Registry^1^ – Universal Trial Number, U1111-1250-1843. Written consent forms to participate in this study were obtained from all patients or substitute decision-makers (for patients lacking decision-making capacity). We retrospectively analyzed the medical history, physical examination, and radiological evaluation results obtained from all patients. Epidemiological, clinical, laboratory, and radiological parameters and treatment, as well as outcome data, were obtained from electronic medical records. The data collection forms were reviewed independently by two researchers. Blood samples were collected on admission and at hospital discharge. For patient #3, who died on the 21st day of hospitalization, blood samples were collected on day 21; and for patient #15, who died on day 17 of hospitalization, blood samples were collected on day 14. For the two deceased patients, length of stay (LOS) was considered 28 days (maximum LOS predicted in the study). Hematological and biochemical parameters in blood samples were determined using automated methods.

### Definition of Clinical Status

According to the original study, all 30 patients with COVID-19 were symptomatic at hospital admission and typical pneumonia was confirmed by computed tomography of the chest and scored (CT score) by two lung expert radiologists, according to parameters described elsewhere (21). COVID-19 diagnosis was confirmed by SARS-CoV-2 RT-PCR and the oxygen saturation measured was ≤94% at ambient air or Pa02/FiO2 ≤ 300 mm Hg. In addition, considering LOS, patients were divided into two groups: severe and critical, according to a definition published elsewhere (20, 22, 23). Patients with LOS for up to 5 days were defined as severe (*n* = 11) and patients with LOS ≥ 13 days were defined as critical (*n* = 12; Supplementary Table 1). Patients with LOS between 6–12 days were not further evaluated in this study (*n* = 7).

### Determination of Serum Parameters

ELISA methods were used to determine triggering receptor expressed on myeloid cells-1 (TREM-1, RD Systems; catalog number DTRM10C), lactate dehydrogenase (MyBioSource; catalog number MBS162869), and serum IgG against SARS-CoV-2 spike protein (Cell Signaling; catalog number 20154). Albumin was determined using a colorimetric assay (LaborLab; catalog number 1770010). C-reactive protein (CRP) was determined using an ELISA kit (Thermo Fisher; catalog number KHA0031). Cytokines were determined using multiplex Human Cytokine 25-Plex Panel (Invitrogen; catalog number LHC0009) and ELISA kits (Interleukin-1beta, RD Systems; catalog number HSLB00C; IL-6, RD Systems; catalog number D6050; eotaxin-2, RayBiotech; catalog number O00175).

### Determination of Angiotensin Pathway Components

ELISA kits were also used to measure ACE (ACE MyBioSource; catalog number MBS2700706), ACE2 (ACE 2 MyBioSource; catalog number MBS2703852), angiotensin (1–7; ANG 1-7 MyBioSource; catalog number MBS2607668), angiotensin (1–9; ANG 1–9 MyBioSource; catalog number MBS162788), and angiotensin 2 (ANG II MyBioSource; catalog number MBS7003599).

### Real-Time Reverse Transcription PCR Assay

Gene expression of cytokines and immune cell molecular markers were determined by RT-PCR. Blood was collected using the PAXgene^®^ Blood RNA tube and frozen at –80^°^C for further analysis. Total RNA from whole blood was extracted and purified using PAXgene Blood RNA Kit (PreAnalytix; catalog number 762174). cDNA samples were obtained from 600 ng of RNA using High-Capacity cDNA Reverse Transcription Kit (Applied Biosystems; catalog number 4368813). Gene expression analyses were performed using the StepOne™ Real-Time PCR System (Applied Biosystems). Each PCR well contained 5 ng of cDNA, 0.25 μl of specific primer, 3.0 μl of Taqman Universal master mix (Applied Biosystems; catalog number 4369016), and RNase-free water to 7.5 μl of the final volume. Primers were purchased from Applied Biosystems: *IL6* – Hs00985639_m1; *CXCL8* – Hs00174103_m1; *CCL2* – Hs00234140_m1; *TNF*α – Hs00174128_m1; *IL10* – Hs00961622_m1; *IL1*β – Hs0155410_m1; *CCR3 –* Hs00266213_m1; *TLR7* – Hs01933259_m1; *CD11B* – Hs00167304_m1; *CD15* – Hs1106466_s1; *CD66b* – Hs00266198_m1; *IL5RA* – Hs00602482_m1; and *CD16A* – Hs02388314_m1. Human *Gapdh* (Cat. Number 4326317E), and *PPIA* (Hs04401210_s1) were employed in all samples as normalizing transcripts. PCR efficiency was calculated before the experiments, and the 2^-DDCT method was used to calculate gene expression.

### Statistical Analysis

All statistical analyses were performed using GraphPad Prism 8.0 and SPSS version 22.0. Values are presented as mean and standard deviation (SD) in the tables and means and standard error of the mean in the figures. Before the analyses, a Kolmogorov–Smirnov test was used to evaluate the normal distribution of variables. If the variable had non-parametric distribution, the equivalent non-parametric test was used. For most analyses, three different variables associated with disease severity were considered: total duration of the disease, LOS (hospitalization), and length of intensive care. Associations between duration of the disease, LOS, and length of intensive care with clinical parameters and biochemical markers were performed using Spearman or Person coefficient. A *p*-value < 0.05 was considered significant.

## Results

### Clinical, Laboratorial, and Radiological Characteristics of Patients at Admission

Supplementary Table 1 presents the age, sex, date of hospital admission, LOS, and disease severity of each patient included in the study. Baseline clinical and laboratorial data are presented in Table 1. Compared to severe patients, critical patients presented higher CT scores, lower diastolic blood pressure, higher blood lactate dehydrogenase, and higher CRP. There were no differences in comorbidities among the groups (Supplementary Table 2).

**TABLE 1:** Clinical and laboratorial parameters at inclusion.

Parameter	All patients	Severe	Critical	*P-value*
Female/male	14/16	4/7	5/7	
Age (y)	51.6 ± 11.5	55.7 ± 3.1	49 ± 3.2	0.07
BMI (kg/m^2^)	30.6 ± 6.7	29 ± 2.0	31.8 ± 2.3	0.36
Symptoms onset (d)	8.1 ± 2.5	8.1 ± 0.6	8.2 ± 0.7	0.75
SatO2 (%)	91.4 ± 4.7	94.8 ± 1.0	95.6 ± 2.9	0.58
Lung CT score	17.8 ± 7.3	13.2 ± 1.6	22.7 ± 1.6	<0.01
Systolic blood pressure (mm Hg)	124 ± 18	134.4 ± 5.6	121 ± 2.7	0.08
<90 mm Hg (*n*)	*1 (3.3%)*	*0 (0%)*	*1 (8.3%)*	
Diastolic blood pressure (mm Hg)	76.7 ± 12.7	86.9 ± 2.7	72.0 ± 3.0	<0.01
<60 mm Hg (*n*)	*0 (0%)*	*0 (0%)*	*0 (0%)*	
White cell count (×10^9^/L)	7.76 ± 4.0	7.0 ± 1.3	8.4 ± 0.9	0.24
<4 × 10^9^/L (*n*)	*5 (16.7%)*	*3 (27.3%)*	*1 (8.3%)*	
4–10 × 10^9^/L (*n*)	20 (66.7%)	7 (63.6%)	8 (66.7%)	
>10 × 10^9^/L (*n*)	*5 (16.7%)*	*1 (9%)*	*3 (25%)*	
Lymphocyte count (×10^9^/L)	1.18 ± 0.48	1.13 ± 0.2	1.15 ± 0.1	0.41
<1 × 10^9^/l (*n*)	*9 (33.3%)*	*4 (44.4%)*	*4 (36.4%)*	
>1 × 10^9^/l (*n*)	*18 (66.7%)*	*5 (55.6%)*	*7 (63.6%)*	
Platelet count (×10^9^/L)	221.7 ± 93.4	214.2 ± 38.9	199.3 ± 16.5	0.79
<100 × 10^9^/L (*n*)	*1 (3.3%)*	*1 (9.1%)*	*0 (0%)*	
≥100 × 10^9^/L (*n*)	*29 (96.7%)*	*10 (90.9%)*	*12 (100%)*	
Plasma glucose (mg/dL)	172.7 ± 96.2	125.6 ± 16.4	145.1 ± 10.6	0.06
<125 (mg/dL; *n*)	*13 (43.3%)*	*8 (72.7%)*	*5 (41.7%)*	
>125 (mg/dL; *n*)	*17 (56.7%)*	*3 (27.3%)*	*7 (58.3%)*	
Serum creatinine (mg/dL)	1.00 ± 0.61	0.87 ± 0.07	1.23 ± 0.26	0.34
<1.2 (mg/dL; *n*)	*26 (86.7%)*	*10 (90.9%)*	*9 (75%)*	
>1.2 (mg/dL; *n*)	*4 (13.3%)*	*1 (9.1%)*	*3 (25%)*	
AST (U/L)	61.8 ± 62.2	74.7 ± 29.3	55.17 ± 5.5	0.53
<40 (U/L; *n*)	*11 (37.9%)*	*5 (50%)*	*2 (16.7%)*	
>40(U/L; *n*)	*18 (62.1%)*	*5 (50%)*	*10 (83.3%)*	
ALT (U/L)	48.3 ± 52.7	66.4 ± 25.6	38.3 ± 4.9	0.63
<40 (U/L; *n*)	*17 (58.6%)*	*6 (60%)*	*6 (50%)*	
≥40(U/L; *n*)	*12 (41.4%)*	*4 (40%)*	*6 (50%)*	
LDH (U/L)	362.2 ± 176.0	287.2 ± 21.3	477.6 ± 70.2	<0.01
<245 (U/L; *n*)	*5 (17.9%)*	*4 (36.4%)*	*1 (9.1%)*	
≥245 (U/L; *n*)	*23 (82.1%)*	*7 (63.6%)*	*10 (90.9%)*	
C-Reactive Protein (mg/L)	117.6 ± 74.3	74.7 ± 11.6	154.1 ± 24.5	<0.05
<10 (mg/L; *n*)	*0 (0%)*	*0 (0%)*	*0 (0%)*	
≥10 (mg/L; n)	*29 (100%)*	*11 (100%)*	*12 (100%)*	

*ALT, alanine aminotransferase; AST, aspartate aminotransferase; BMI, body mass index; CK, creatine kinase; and LDH, lactate dehydrogenase. Unpaired Student t-test was used to compare Severe × Critical conditions.*

### Defining the Clinical, Laboratorial, and Radiological Parameters Associated With Poor Outcomes

**FIGURE 1:**
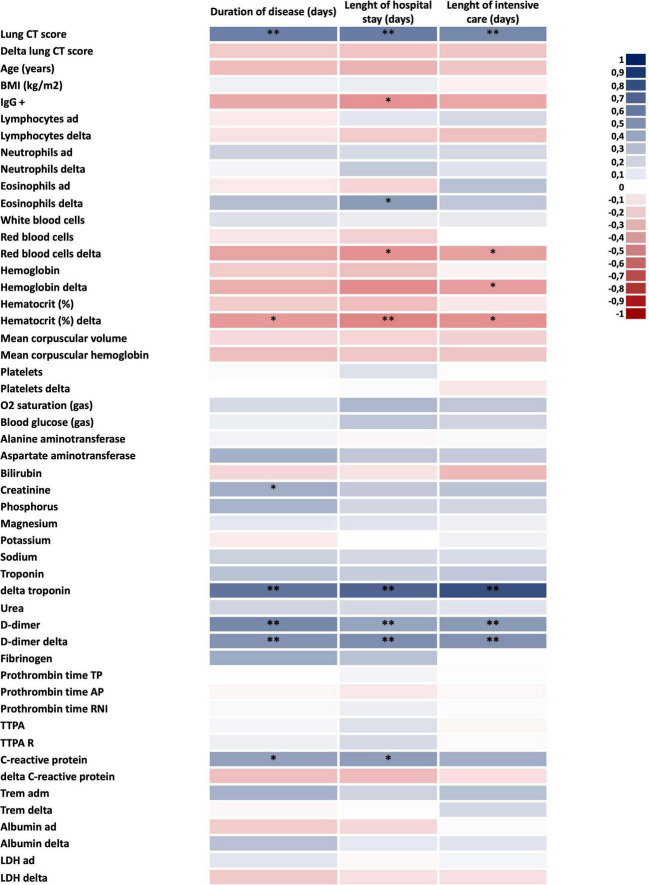
Defining the factors associated with COVID-19 severity. Heatmap of clinical and laboratorial parameters potentially associated with COVID-19 severity. Blue colors mean direct correlation; red colors mean inverse correlation. In all *n* = 28; *p < 0.05 and ^**^p < 0.01. BMI, body mass index; CT, computed tomography; ICU, intensive care unit; LDH, lactate dehydrogenase; and TTPA, partial activated thromboplastin time.

To validate this cohort as representative of the expected outcomes of patients with severe COVID-19, we measured several parameters and evaluated their association with poor prognosis, defined by the number of days from the beginning of symptoms to hospital discharge; the number of days in hospital; and, the number of days in the intensive care unit. As depicted in Figure 1, admission CT score, variation in delta-troponin, baseline D-dimer, change in D-dimer during hospitalization, and baseline CRP were positively correlated with poorer prognosis. In addition, baseline creatinine was positively correlated with total disease time, whereas the change in blood eosinophils correlated with time in hospital. Conversely, variation in red blood cell counts and hematocrit variation were negatively correlated with prognosis.

### Blood Cytokines/Chemokine Associated With Poor Prognosis

**FIGURE 2:**
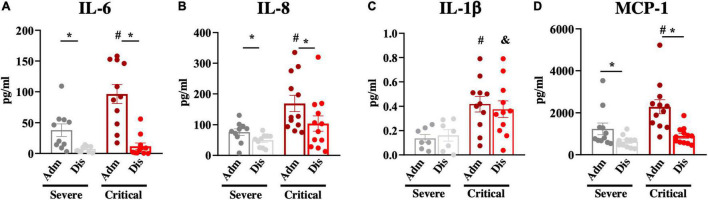
Blood inflammatory markers. Inflammatory markers; interleukin-6 (IL-6; **A**), interleukin-8 (IL-8; **B**), interleukin-1beta (IL-1β; **C**), and monocyte chemoattractant protein-1 (MCP-1; **D**) were determined in blood samples at hospital admission (Adm) and discharge (Dis). *p < 0.05; #*p* < 0.05 vs. severe at admission; &*p* < 0.05 vs. severe at discharge.

We further validated the cohort by determining the blood levels of three cytokines and one chemokine known to be increased in the blood of patients with severe COVID-19. In Figure 2, we show that admission blood IL-6, IL-8, IL-1β, and monocyte chemoattractant protein-1 were significantly higher in patients with poorer prognoses.

### Admission Plasma Angiotensin II Levels Predict Critical Coronavirus Disease 2019

**FIGURE 3:**
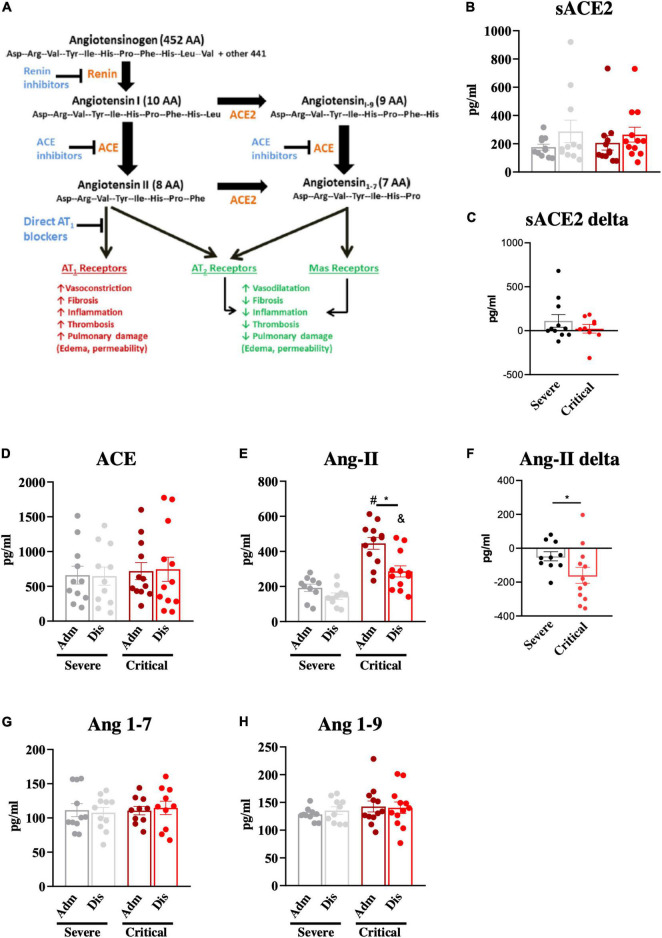
Components of the renin-angiotensin-aldosterone system. The renin-angiotensin-aldosterone system and its main effects are depicted in **(A)**. Components of the renin-angiotensin-aldosterone system; soluble angiotensin-converting enzyme-2 (sACE2; **B, C**), angiotensin-converting enzyme (ACE; **D**), angiotensin-II (Ang-II; **E, F**), angiotensin 1–7 (Ang 1–7; **G**) and angiotensin 1–9 (Ang 1–9; **H**) were determined in blood samples at hospital admission (Adm) and discharge (Dis). The variations of sACE2 **(C)** and Ang-II **(F)** from admission to discharge were determined. *p < 0.05; #*p* < 0.05 vs. severe at admission; &*p* < 0.05 vs. severe at discharge.

Next, we determined the plasma levels of key components of the RAAS (Figure 3A) at admission and discharge. There were no significant differences in soluble ACE2 (Figure 3B), as well as in the variation of soluble ACE2, from admission to discharge (Figure 3C). Plasma ACE levels were also similar in both groups and underwent no changes from admission to discharge (Figure 3D). Conversely, admission Ang-II levels were significantly higher (approximately 2-fold higher) in patients presenting with critical COVID-19 compared to patients presenting with severe disease (Figure 3E). Moreover, there was a significantly greater decrease in Ang-II plasma levels from admission to discharge in critical patients compared to severe patients (Figure 3F). Importantly, all patients presenting Ang-II plasma levels greater than 300 pg/ml presented a critical outcome of the disease. There were no differences in the plasma levels of Ang (1–7; Figure 3G) and Ang (1–9; Figure 3H) between the groups. As the existence of comorbidities could represent a bias in the analysis, we evaluated RAAS components by comparing patients with and without comorbidities. As shown in Supplementary Table 3, only Ang (1–7) was different among the groups presenting higher levels at admission in patients without comorbidities.

## Discussion

This study shows that at hospital admission, plasma levels of Ang-II are higher in patients with COVID-19 presenting with the critical disease compared to patients presenting with severe disease. This data indicates Ang-II may be a potential biomarker that could help identify hospitalized patients with poorer COVID-19 prognosis and, also stimulate further research aimed at developing therapeutic strategies to mitigate disease severity. It is important to highlight that other biomarkers have been previously identified and seem to be associated with the pathological process of COVID-19. The incorporation of Ang-II to this list could help to understand the mechanisms leading to severe forms of the disease, as well as provide an advance in the capacity of predicting critical outcomes and the early need for intensive care (24).

The patients included in the study were ranked for severity based on the LOS. We decided for using this approach because, as a retrospective study that asked if admission levels of components of the RAAS could be good biomarkers for predicting the outcomes of severe COVID-19, we reasoned that upon employment of other known markers of severity, such as blood pressure, oxygen requirements, and imaging progression, we could generate a bias in the interpretation of the results that could mask the real value of RAAS components in the context.

Despite the undisputed importance of studying the RAAS in COVID-19, there are only few studies that have actually measured the components of this system in patients. Most of the studies have simply compared non-infected vs. SARS-CoV-2-infected patients without considering disease severity and clinical outcomes. In this context, two studies reported no differences in the blood levels of Ang-II (18, 25) and one study reported a reduction of Ang-II and an increase in angiotensin (1–7; 26). It is currently known that SARS-CoV-2 infection can lead to a large spectrum of clinical outcomes that range from sore throat, mild fever, and tiredness to severe lung damage, cardiovascular dysfunction, and coagulopathy (22, 23). Approximately 81% of patients with COVID-19 present with mild or moderate disease and are not admitted for hospital care (27); conversely, of the remaining symptomatic patients that require hospitalization, three-fourths have severe disease and one-fourth have critical disease (27). The studies that have evaluated the components of the RAAS, comparing SARS-CoV-2-infected vs. non-infected patients, could provide important mechanistic advances if they showed that SARS-CoV-2 infection, independently of severity, could modulate the components of the RAAS. In at least one study, the treatment of rodents with purified spike protein induced an increase in Ang-II, providing an interesting experimental proof-of-concept to support the hypothesis that SARS-CoV-2 binding to ACE2 can indeed impact the regulation of the RAAS (28, 29). However, the few clinical studies available to date present conflicting results. In some studies, there were methodological issues, such as small sample size and inappropriate assays for measuring RAAS peptides; and in others, statistical power was insufficient to confirm or refute the impact of SARS-CoV-2 infection on the RAAS. Thus, further studies should be performed in order to provide a definitive answer to this important question.

Identifying biomarkers that are modulated upon SARS-CoV-2 infection, independently of disease severity, is important and could provide advances in the understanding of the mechanisms of disease, but they have limited value in population screening, as the majority of infected patients have either no or very mild symptoms and do not require medical intervention (23). This is the case for IL-6, TNF-α, IL-1β, and IL-10, which present early increases in the blood of patients that will progress with severe disease as compared with patients presenting only mild symptoms that require no hospitalization (30). Conversely, identifying biomarkers that segregate hospitalized patients who present with severe or critical diseases could provide therapeutic advances, as patients at greater risk could be given intensive care promptly upon admission. Prior to this study, there were only two reports on the measurement of the components of the RAAS in the plasma of patients with COVID-19 presenting with distinct clinical statuses. One study compared patients during an early phase of COVID-19 manifestations vs. patients with prolonged disease (31). The authors reported a reduction of ACE2 expression in cells and an increase in the blood level of Ang-II in patients presenting with prolonged disease (31). Another study evaluated patients with severe COVID-19 vs. uninfected subjects (17), documenting an increase in the blood levels of Ang-II in severe patients, and particularly, a positive correlation between blood Ang-II and viral load (17). Of note, the blood levels of Ang-II in severe patients in this study were similar to the levels (approximately 400 pg/ml) we found in our study of patients with critical COVID-19.

Despite the advance provided by this study, there are weaknesses that could be addressed in future work: (i) the number of patients is small, but it is still similar to other previous studies that evaluated the RAAS in patients with COVID-19 (18, 25, 26, 32); (ii) as this is a retrospective study, we could not determine the real-time correlation between the rate of clinical improvement and changes in the blood levels of RAAS components; (iii) as the study was performed in the very beginning of COVID-19 pandemic, we were not equipped to perform determination of viral load or spike protein amount; and (iv) there are unsolved methodological critics regarding the accuracy of the available Ang-II determination methods (32–34).

In conclusion, blood levels of angiotensin-II are increased in hospitalized patients with COVID-19 presenting the critical outcome of the disease. This could place Ang-II alongside D-dimer (35), ferritin (36), CRP (37), IL-6, and tumor necrosis factor-alpha (38) as early biomarkers for prognosis and, therefore, with the ability to guide the clinical decision.

## Data Availability Statement

The raw data supporting the conclusions of this article will be made available by the authors, without undue reservation.

## Ethics Statement

The studies involving human participants were reviewed and approved by Ethics Committee of the University of Campinas. The patients/participants provided their written informed consent to participate in this study.

## Author Contributions

LV and AS are the supervisors and lead contacts, conceived the study, raised funds, organized the protocol, and wrote the manuscript. RC and BB performed laboratory analysis. MM-P and EA organized data. EM coordinated the clinical team. AP, LR, RU, AB, TN, and MA screened, enrolled, and followed-up patients. MM provided clinical supervision support for the preparation and execution of the trial. RD and SD designed and performed radiological analysis. MM-P performed the statistical analysis. All authors read and approved the final manuscript.

## Conflict of Interest

The authors declare that the research was conducted in the absence of any commercial or financial relationships that could be construed as a potential conflict of interest.

## Publisher’s Note

All claims expressed in this article are solely those of the authors and do not necessarily represent those of their affiliated organizations, or those of the publisher, the editors and the reviewers. Any product that may be evaluated in this article, or claim that may be made by its manufacturer, is not guaranteed or endorsed by the publisher.

## Footnotes

1
https://ensaiosclinicos.gov.br/rg/RBR-5s2mqg
