# Supplementary material for: Plasma Angiotensin II Is Increased in Critical Coronavirus Disease 2019
Source: Front Cardiovasc Med. 2022 Jun 24;9:847809. doi: 10.3389/fcvm.2022.847809 (PMC9263116; doi:10.3389/fcvm.2022.847809)
Supplement: Supplementary file 1 [file Data_Sheet_1.docx]

**Supplementary Data**

**Plasma angiotensin II is increased in critical COVID-19**

Rafael L. Camargo et al

**Supplementary Table 1. Patients included in the study.**

| **Patient** | **Age** | **Gender** | **Inclusion** | **Length of stay** | **Status** |
| --- | --- | --- | --- | --- | --- |
| 1 | 50 | M | 2020-04-23 | 5 | Severe |
| 2 | 57 | M | 2020-04-24 | 4 | Severe |
| 3 | 74 | F | 2020-04-29 | 28^*^ | Critical |
| 5 | 47 | M | 2020-05-01 | 28^*^ | Critical |
| 6 | 57 | M | 2020-05-04 | 4 | Severe |
| 7 | 47 | M | 2020-05-07 | 14 | Critical |
| 8 | 47 | M | 2020-05-14 | 28 | Critical |
| 9 | 38 | M | 2020-05-15 | *25* | Critical |
| 10 | 53 | M | 2020-05-19 | 13 | Critical |
| 11 | 62 | F | 2020-05-19 | 4 | Severe |
| 14 | 50 | M | 2020-05-26 | 5 | Severe |
| 15 | 49 | F | 2020-05-26 | 17 | Critical |
| 16 | 43 | M | 2020-05-27 | 4 | Severe |
| 17 | 54 | F | 2020-05-27 | 5 | Severe |
| 18 | 46 | F | 2020-05-27 | 28 | Critical |
| 20 | 65 | M | 2020-05-28 | 4 | Severe |
| 21 | 40 | M | 2020-05-29 | 4 | Severe |
| 23 | 43 | F | 2020-06-04 | 18 | Critical |
| 24 | 36 | M | 2020-06-04 | 13 | Critical |
| 25 | 42 | F | 2020-06-08 | 21 | Critical |
| 27 | 77 | F | 2020-06-10 | 5 | Severe |
| 28 | 66 | F | 2020-06-12 | 18 | Critical |
| 30 | 58 | F | 2020-06-14 | 5 | Severe |

*Patients died as a result of COVID-19; length of stay was defined as 28 days

**Supplementary Table 2. Comorbidities of patients at admission.**

| *Comorbidities at baseline* | *All patients*  *(n:30)* | *Severe*  *(n:11)* | *Critical*  *(n:12)* | *p-Value* |
| --- | --- | --- | --- | --- |
| *Hypertension, n (%)* | 15 (50) | 5 (45.5) | 6 (50) | 0.89 |
| *Diabetes Mellitus, n (%)* | 14 (46.7) | 4 (36.4) | 5 (41.7) | 0.31 |
| *Obesity, n (%)* | 14 (46.7) | 3 (27.3) | 7 (58.3) | 0.32 |
| *Dislipidemia, n (%)* | 5 (16.7) | 2 (18.2) | 1 (8.3) | 0.51 |
| *Former smoker, n (%)* | 4 (13.3) | 2 (18.2) | 1 (8.3) | 0.77 |
| *Hypothyroidism, n (%)* | 2 (6.7) | 1 (9.1) | 0 (0) | 0.45 |
| *Asthma, n (%)* | 1 (3.3) | 0 (0) | 1 (8.3) | 0.46 |
| *Smoker, n (%)* | *1 (3.3)* | *0 (0)* | *1 (8.3)* | *0.46* |
| *Fibromyalgia, n (%)* | 1 (3.3) | 0 (0) | 1 (8.3) | 0.46 |

Chi-square test was employed to analyze parameters.

**Supplementary Table 3.**

|  | Without Comorbidities | With Comorbidities | p-Value |
| --- | --- | --- | --- |
| Patients (n) | 6 | 24 | . |
| Ang-II (pg/mL) | 355.34 ± 200.87 | 299.95 ± 163.7 | 0.57 |
| ACE (pg/mL) | 671.23 ± 424.54 | 664.94 ± 449.89 | 0.94 |
| ACE2 (pg/mL) | 162.96 ± 85.67 | 203.17 ± 132.86 | 0.30 |
| A1-7 (pg/mL) | 136.94 ± 29.56 | 103.25 ± 21.8 | 0.02 |
| A1-9 (pg/mL) | 244.05 ± 235.81 | 134.52 ± 19.76 | 0.49 |
